# Resistance to preservatives and the viable but non-culturable state formation of *Asaia lannensis* in flavored syrups

**DOI:** 10.3389/fmicb.2024.1345800

**Published:** 2024-02-16

**Authors:** Xia Wen, Yiwen Chen, Shuyao Zhang, Ai-ting Su, Di Huang, Gang Zhou, Xiaobao Xie, Jufang Wang

**Affiliations:** ^1^School of Biology and Biological Engineering, South China University of Technology, Guangzhou, China; ^2^Guangdong Provincial Key Laboratory of Fermentation and Enzyme Engineering, South China University of Technology, Guangzhou, China; ^3^Key Laboratory of Agricultural Microbiomics and Precision Application (Ministry of Agriculture and Rural Affairs), Guangdong Provincial Key Laboratory of Microbial Culture Collection and Application, Key Laboratory of Agricultural Microbiome (Ministry of Agriculture and Rural Affairs), State Key Laboratory of Applied Microbiology Southern China, Guangdong Detection Center of Microbiology, Institute of Microbiology, Guangdong Academy of Sciences, Guangzhou, China

**Keywords:** *Asaia lannensis*, resistance, VBNC, preservatives, flavored syrups

## Abstract

Food security is a crucial issue that has caused extensive concern, and the use of food flavors has become prevalent over time. we used the molecular biological techniques, preservative susceptibility testing, viable but non-culturable (VBNC) state induction testing, and a transcriptome analysis to examine the bacterial contamination of favored syrup and identify the causes and develop effective control measures. The results showed that *Asaia lannensis* WLS1-1 is a microorganism that can spoil food and is a member of the acetic acid bacteria families. The minimum inhibitory concentration (MIC) and minimum bactericidal concentration (MBC) tests showed that WLS1-1 was susceptible to potassium sorbate (PS), sodium benzoate (SB), and sodium sulffte (SS) at pH 4.0. It revealed a progressive increase in resistance to these preservatives at increasing pH values. WLS1-1 was resistant to PS, SB and SS with an MIC of 4.0, 2.0 and 0.5  g/L at pH 5.0, respectively. The MIC values exceed the maximum permissible concentrations that can be added. The induction test of the VBNC state demonstrated that WLS1-1 lost its ability to grow after 321 days of PS induction, 229  days of SB induction and 52  days of SS induction combined with low temperature at 4°C. Additionally, laser confocal microscopy and a propidium monoazide-quantitative polymerase chain reaction (PMA-qPCR) assay showed that WLS1-1 was still alive after VBNC formation. There were 7.192 ± 0.081 (PS), 5.416 ± 0.149 (SB) and 2.837 ± 0.134 (SS) log_10_(CFU/mL) of viable bacteria. An analysis of the transcriptome data suggests that Asaia lannensis can enter the VBNC state by regulating oxidative stress and decreasing protein synthesis and metabolic activity in response to low temperature and preservatives. The relative resistance of Asaia lannensis to preservatives and the induction of the VBNC state by preservatives are the primary factors that contribute to the contamination of favored syrup by this bacterium. To our knowledge, this study represents the first evidence of the ability of *Asaia lannensis* to enter the VBNC state and provides a theoretical foundation for the control of organisms with similar types of activity.

## 1 Introduction

The genus *Asaia* was isolated from flowers and fermented glutinous rice in Indonesia and Thailand in 2000 and belongs to the acetic acid bacterial lineage of the Acetobacteraceae family. Currently, this family is composed of eight species, including *Asaia bogorensis*, *Asaia siamensis*, *Asaia krungthepensis*, *Asaia astilbis*, *Asaia lannensis*, *Asaia platycodi*, *Asaia prunellae*, and *Asaia spathodeae*, which were first isolated from tropical flowers (Table 1). Furthermore, these bacteria are catalase-positive and capable of oxidase-negative fermentation. They differ from the other acetic acid bacteria because they do not promote the production of acetic acid from ethanol and do not grow in the presence of 0.35% (v/v) acetic acid. These bacteria are resistant to pasteurization during food processing and can cause food to spoil during the shelf life under normal packaging and storage conditions, which hampers the prevention and control of contaminants.

**Table 1:** Eight reported species of *Asaia.*

Species	Source	Country	References
*Asaia bogorensis*	Flower of an orchid tree (*Bauhinia purpurea*)	Indonesia	Yamada et al. (2000)
*Asaia siamensis*	Tropical flowers	Thailand	Katsura et al. (2001)
*Asaia krungthepensis*	Heliconia flowers	Bangkok, Thailand	Yukphan et al. (2004)
*Asaia lannensis*	Spider lily flowers	Chiang Mai, Thailand	Malimas et al. (2008)
*Asaia spathodeae*	African tulip flowers	Thailand	Kommanee et al. (2010)
*Asaia astilbes*	Flowers	Japan	Suzuki et al. (2010)
*Asaia platycodi*	Flowers	Japan	Suzuki et al. (2010)
*Asaia prunellae*	Flowers	Japan	Suzuki et al. (2010)

Two species, *Asaia bogorensis* and *Asaia lannensis*, have previously been reported to be involved in spoiled food. For example, some reports demonstrated that *Asaia bogorensis* and *Asaia lannensis* were isolated from fruit-flavored bottled water (>10^6^ CFU/mL) (Moore et al., 2002; Sedláčková et al., 2011), fruit drinks and ice teas (Horsáková et al., 2009), and strawberry-flavored bottled water (Kregiel et al., 2012). These drinks are primarily composed of concentrates of natural fruit flavors, sugars, organic acids, and preservatives. Therefore, microbial species that tolerate these conditions may survive and grow. In addition, some studies demonstrated that these bacteria could be opportunistic pathogens in patients with reduced immunity, such as bloodstream infection after bone marrow transplantation (Snyder et al., 2004) and bacteremia in cases of intravenous drug use (Tuuminen et al., 2006; Abdel-Haq et al., 2009). *Asaia lannensis* was also detected in nosocomial infections in pediatric patients with idiopathic dilated cardiomyopathy (Juretschko et al., 2010) and transient bacteremia owing to *Asaia lannensis* in a patient with a psychiatric disorder (Carretto et al., 2016).

Acetic acid bacteria can induce food to spoil and cause clinical-related infections owing to their high resistance to common food preservatives, including chemical preservatives (Horsáková et al., 2009) and antibiotics (Alauzet et al., 2010), and their strong ability to survive in extreme environmental conditions, such as high/low temperatures, drying and irradiation.

Responses to environmental factors, such as temperature, pH, preservatives and glucose, impact not only resistance but also growth and survival (Roy et al., 2021). Some bacteria lose their ability to be cultured on/in nutrient media and are defined as viable but nonculturable (VBNC) (Xu et al., 1982; Colwell et al., 1985). In response to nutrient deficiency, these species can reduce their rate of respiration and density and retain essential metabolic activities (Zhao et al., 2017). To date, 43 foodborne pathogenic bacteria and 14 spoilage/functional microorganisms were identified as being capable of undergoing a VBNC state in food (Dong et al., 2020). For example, wine and beer can spoil owing to the activity of certain bacteria, including *Listeria monocytogenes* (Lotoux et al., 2022), *Salmonella* (Shi et al., 2022), *E. coli* O157:H7 (Wei and Zhao, 2018), *Pseudomonas aeruginosa* (Qi et al., 2022), *Staphylococcus aureus* (Yan et al., 2021), *Bifidobacterium longum* (Lahtinen et al., 2008), *Acetobacter aceti* (Millet and Lonvaud-Funel, 2000), and *Acetobacter senegalensis* (Shafiei et al., 2014). Ravel et al. (1994) first proposed the hypothesis that gene regulation is responsible for the formation of the VBNC state in 1994. Since then, many studies have confirmed this hypothesis and revealed significant changes in various genes, including those involved in transcriptional regulation (Kusumoto et al., 2012), oxidative stress (Morishige et al., 2013), outer membrane proteins (Asakura et al., 2008), metabolism (Nosho et al., 2018), and toxin-related processes (Pedersen et al., 2002). However, there is currently a lack of research on the mechanism used by *Asaia* to regulate genes when it is in the VBNC state.

In this study, bacteria were isolated and identified from flavored syrup that had spoiled. The resistance against conventional preservatives was analyzed; the VBNC state of bacteria was induced by preservatives, and the expression of genes in the VBNC state was evaluated to understand the factors that lead to contamination, which provides targets for prevention and control.

## 2 Materials and methods

### 2.1 Isolation and identification of the spoilage strain

In this study, one bacterial strain *Asaia lannensis* was isolated from spoiled flavored syrup samples and designated WLS1-1. Morphological, physiological, and molecular methods were performed to identify the strain, and the nucleotide sequences of the 16S rRNA genes were deposited in GenBank (NCBI). Finally, the bacterial strain was stored with 20% glycerol at −80°C for further characterization.

### 2.2 Susceptibility of WLS1-1 to food preservatives

The susceptibility of WLS1-1 to some preservatives, including potassium sorbate (PS), sodium benzoate (SB), and sodium sulfite (SS) (Sigma-Aldrich, Shanghai, China) was evaluated. These compounds are frequently utilized in the food industry during the steps of production. Furthermore, the MIC (minimum inhibitory concentration) values were determined using a microdilution broth assay. Briefly, the final concentrations of the preservatives were 32, 16, 8, 4, 2, 1, and 0.5 g/L, respectively. No preservatives were added as negative controls. A volume of 10 μL of each concentration was added to each well to prepare a bacterial solution that contained 10^5^ CFU/mL. Finally, 180 μL of AS medium (composed of 0.5% w/v peptone, 0.5% w/v yeast extract, and 2% w/v glucose) was added to the wells. Next, the plates were incubated for 24 h using a multifunctional microplate reader. Simultaneously, the growth curves were obtained hourly by measuring the OD_600_ at 28°C (Tecan, Männedorf, Switzerland). In this study, the MIC was defined as the lowest concentration of an antimicrobial compound where no bacterial growth was observed as a change in OD_600_ compared to the negative control. Each assay was performed in triplicate, and the results were presented as the mean ± SD. The minimum bactericidal concentrations (MBCs) were then determined after the MIC test. Briefly, 10 μL of the sample was collected from the MIC wells, plated on agar plates, and incubated at 28°C for 24 h. The MBC was defined as the lowest concentration of preservative where no colonies were observed in the agar plates.

### 2.3 Induction of the VBNC state in WLS1-1

**Figure 1:**
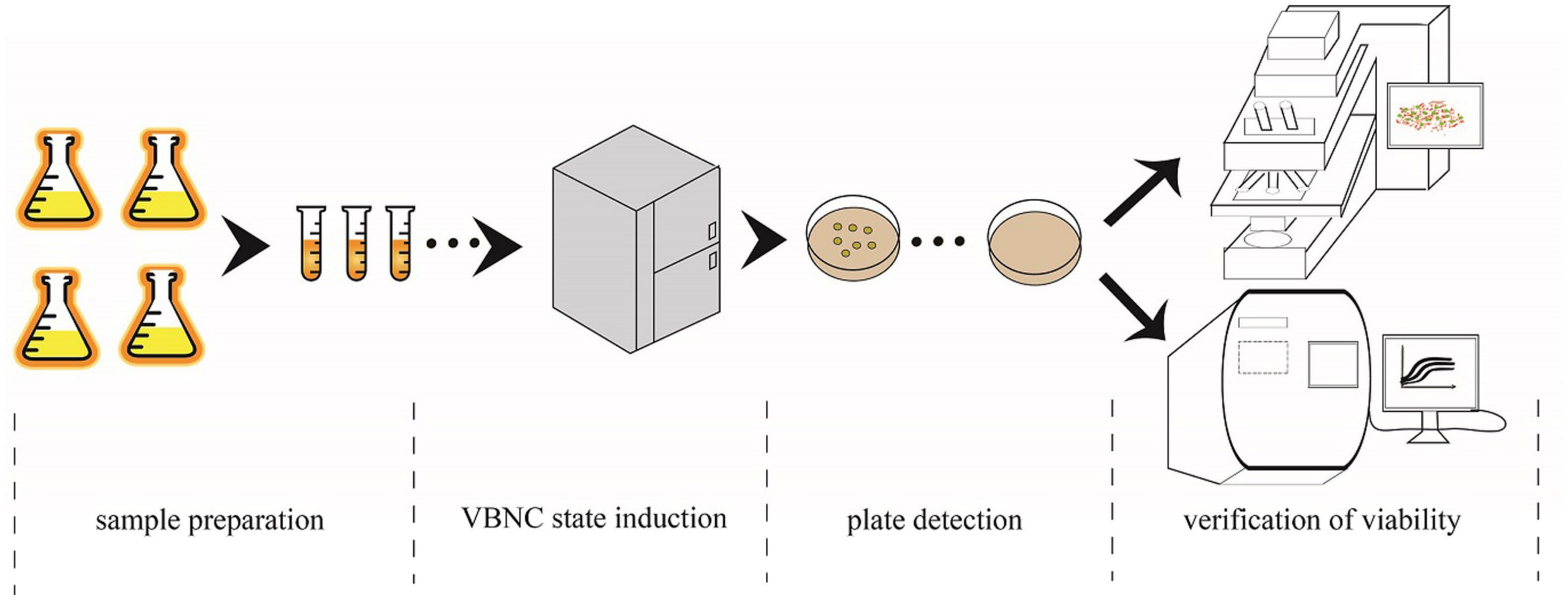
VBNC state induction procedure. Sample preparation: bacteria multiply, preservatives added and subsampled. VBNC state induction: sample placed in the refrigerator at 4°C. Plate detection: samples were taken from the refrigerator every 7 days for testing using the traditional plate culture method. Verification of viability: CLSM detection and PMA-qPCR were used to detect whether the colony survived when no colonies appear on the plates. CLSM, confocal microscopy; PMA-qPCR, propidium monoazide-quantitative PCR; VBNC, viable but non-culturable.

The flow chart of the induction experiment is shown in Figure 1. The preservatives used in this experiment were PS, SB, and SS. First, the WLS1-1 bacterial suspension was inoculated in AS medium (glucose 2% [w/v], yeast extract 0.5% [w/v], and peptone 0.5% [w/v]) at 28°Cand 180 rpm until the mid-logarithmic phase was reached. WLS1-1 was then plated on AS solid medium (AS medium+ agar 1.5% [w/v]) at 28°C for 24 h. Next, a single colony was selected and added to the AS medium, which was adjusted to pH 5.0 with HCl, and a 10^8^ CFU/mL bacterial suspension was prepared and added to an antiseptic solution. In this assay, 1.0 g/L, 1.0 g/L, and 0.05 g/L of PS, SB, and SS, respectively, were used, and their ability to inhibit WLS1-1 was determined according to the GB2760-2014 National Food Safety Standard for the Use of Food Additives in Flavored Syrup. To avoid contamination caused by repeated removal, the bacterial suspension was divided into several tubes (final volume of 1.1 mL) and placed at 4°C for further induction. Finally, the numbers of the bacteria were counted weekly to determine the culturability of WLS1-1.

### 2.4 PMA-qPCR quantitative detection of the VBNC state of WLS1-1

In this experiment, the number of viable cells was counted using propidium monoazide (PMA, Biotium, Inc., Fremont, CA, United States) and quantitative PCR (PMA-qPCR). Briefly, 10 mL of the four groups of VBNC-treated bacteria were added to 100 μL of PMA (at a concentration of 10 μg/mL), and the samples were then incubated at room temperature in the dark for 15 min with continuous agitation. After incubation, the samples were exposed to a 500 W halogen light source with a distance of 20 cm for 5 min on ice with occasional shaking. Next, the samples were centrifuged at 12,000 *g* for 5 min and washed three times with PBS (pH 7.4) to discard the free PMA. In this study, different samples were used, including the preservative-induced WLS1-1 samples, heat-treated samples (treated at 70°C for 5 min and designated as the positive control), and untreated samples (defined as the negative control samples). The bacterial DNA was extracted using a QIAamp DNA Mini Kit (Takara, Kyoto, Japan) according to the manufacturer’s instructions. The qPCR was performed on a QuantStudio Real-Time PCR System (Bio-Rad, Hercules, CA, United States) using SYBR Premix Ex Taq (TaKaRa, Dalian, China). The flowing primers were used to target the single copy gene *lan* of WLS1-1: 5′-TCCTACGGGAGGCAGCAGT-3 (forward) and 5′-GCCCTTTACGCCCAGTCATT-3′ (reverse). To determine the sensitivity or limit of detection (LOD) of the qPCR method, 10-fold serial dilutions of WLS1-1, which ranged between 10^8^ and 10^2^ CFU/mL, were performed. Next, the DNA was extracted, and the last signal of the dilutions determined the LOD. The standard curves were determined by plotting the concentration of WLS1-1 and the Ct values. The following equation was utilized to calculate the amplification efficiency (E) of PMA-qPCR:


$$E=\left[10^{-1/\mathrm{slope}}-1\right]\times 100\%$$

### 2.5 Confocal laser scanning microscopy analysis

#### 2.5.1 Sample processing

For this study, WLS1-1 colonies (1.0 × 10^8^ CFU/mL) were washed with PBS and distributed in six tubes. Three tubes were then used as the positive control, and the remaining tubes were heated at 100°C for 5 min and used as the negative control. In addition, four treated samples were prepared, including no preservatives, PS, SB, and SS. Briefly, these samples were centrifuged at 12,000 *g* for 5 min and stained with 3 μM of PI (Invitrogen, Carlsbad, CA, United States) and 10 μM of SYTO9 (Invitrogen) in 1 × PBS for 15 min in the dark. Finally, the excess dyes were washed, and the samples were added to the glass-bottom cell culture dishes.

#### 2.5.2 Image acquisition and analysis

The bacteria were visualized using a Zeiss LSM 700^®^NLO confocal laser scanning microscope (Zeiss, Oberkochen, Germany). The two dyes used in this experiment were excited at 488 nm (argon laser) and 561 nm (diode laser), and the emitted signals were collected at 505–544 nm and 600–700 nm, respectively. At least three microscopic fields were visualized and captured. Green and red cells represent viable and dead cells stained by SYTO9 and PI, respectively.

### 2.6 RNA extraction and transcriptome sequencing

To investigate the physiological changes in the VBNC-induced *Asaia lannensis* caused by low temperature and preservatives, an RNA-seq transcriptomic analysis was performed in four samples, including cells grown without treatment (AL), cells exposed to 4°C (CK), cells exposed to 4°C and 1.0 g/L SB treatment (BN), and cells exposed to 4°C and 1.0 g/L PS treatment (SL). An explanatory description of the sequencing sample is shown in Supplementary Figure S1. The total RNA was extracted using TRIzol (Invitrogen/Life Technologies, Carlsbad, CA, United States) according to the manufacturer’s instructions. The cDNA was then obtained using reverse transcription and enriched by PCR to construct the final sequencing cDNA library. The quality was evaluated using an Agilent 2,100 Bioanalyzer (Agilent Technologies, Santa Clara, CA, United States) and an ABI Step One Plus Real-Time PCR system (Applied Biosystems, Waltham, MA, United States). The cDNA was sequenced using a HiSeq^™^ 2000 Sequencing System (Illumina, San Diego, CA, United States) with single-end technology in a single run at the Beijing Genome Institute (BGI, Shenzhen, China). Finally, some parameters, including the conversion of images to sequences, base-calling, and quality value calculations, were conducted by Illumina GA Pipeline software (version 1.6).

To perform the RNA-seq analysis, the raw reads were cleaned to remove low-quality reads using Cutadapt software (version 1.9.1). In this study, the DESeq package v. 1.6.3 software was applied to detect differentially expressed genes (DEGs) in the sample pairs. To determine significant differences in gene expression, the *p*-value threshold was defined according to the false discovery rate (FDR) < 0.05 and the absolute value of log_2_ fold-change with fragments per kilobases per million mapped reads (FPKM) > 1. Next, the DEGs were subjected to an enrichment analysis using Gene Ontology (GO) and Kyoto Encyclopedia of Genes and Genomes (KEGG) pathways.

### 2.7 Statistical analysis

The results were analyzed and plotted using GraphPad Prism version 6.0 (GraphPad, San Diego, CA, United States). The results were expressed as the mean ± standard deviation (SD). Significant differences were determined using a one-way analysis (ANOVA) with SPSS 17.0 (SPSS, Inc., Chicago, IL, USA). A *p*-value of *p* < 0.05 indicated significant differences, and *p* > 0.05 indicated nonsignificant differences (NS).

## 3 Results

### 3.1 Isolation and identification of the bacterial strains

**Figure 2:**
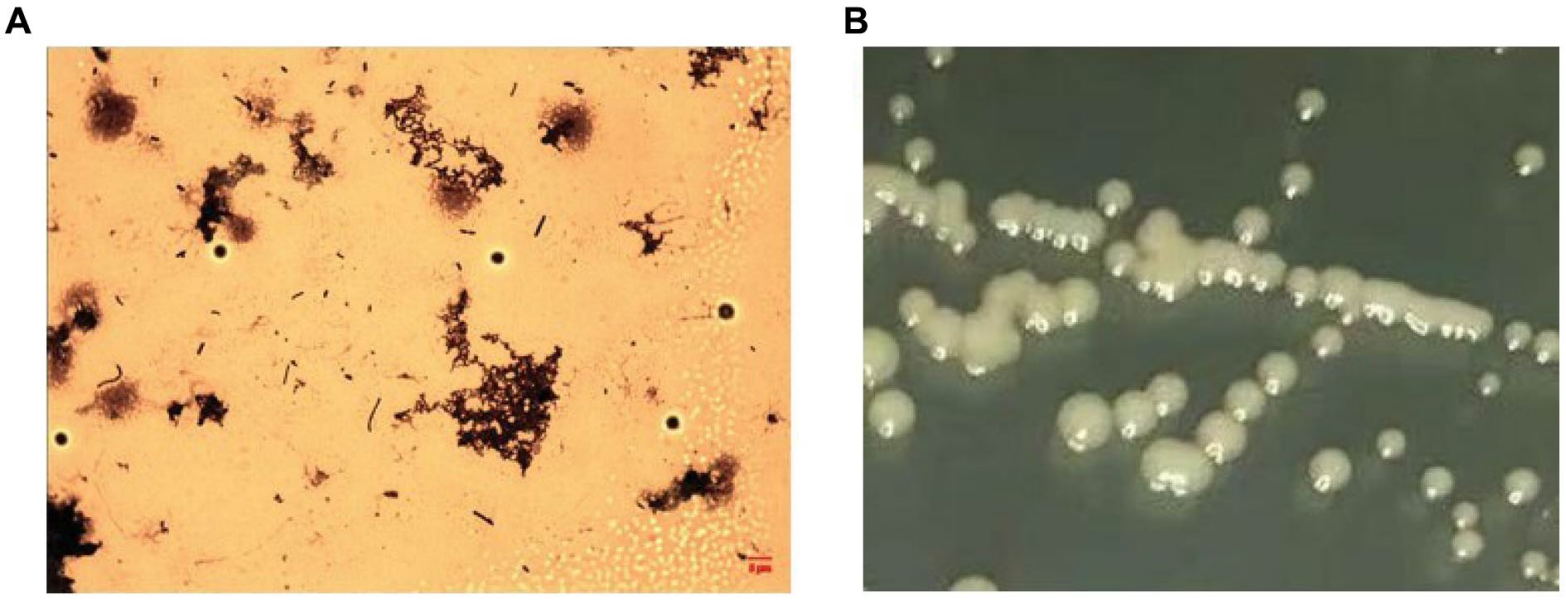
Colony morphology of WLS1-1 in the samples **(A)**, Light micrographs (×1,000, OMAX) and on AS agar plates **(B)**.

After receiving a batch of flavored syrup, a pungent sour odor was evident after opening the pack, and a macroscopic brown flocculent precipitate was observed. Additionally, the pH of sample was lower (3.5) than that of a normal one (5.25). The flocculent precipitate was then collected and placed on a PCA plate at 37°C for more than 7 days. No colonies were observed after incubation, but there was a complete rod-shaped bacterial structure in the flocs (Figure 2A). However, the composition of the medium was changed by adding glucose and adjusting the pH to 5.25 and temperature to 28°C, and a smooth surface that contained small, milky white, round colonies was observed (Figure 2B). The sequencing results obtained from an analysis of the 16S rRNA sequence indicated the presence of an *Asaia* sp. bacterium (with a max identity of 99.87%). An evolutionary tree demonstrated that WLS1-1 was *Asaia lannensis* (Supplementary Figure S2), and the gene number KT596728.1 was obtained after uploading the 16S rRNA sequence to the NCBI database.

### 3.2 Susceptibility of WLS1-1 to food preservatives

In this study, the MICs of preservatives were determined by a standard 2-fold dilution method, and Table 2 shows the MIC and MBC values against the preservatives usually applied in the food industry. Furthermore, the growth curves of *Asaia lannensis* using diverse concentrations of preservatives at different pH values are shown in Supplementary Figure S3.

**Table 2:** The MIC and MBC values of the three preservatives to *Asaia lannensis* at different pH values.

Preservatives	MIC (g/L)				MBC (g/L)			
	pH = 4	pH = 5	pH = 6	pH = 7	pH = 4	pH = 5	pH = 6	pH = 7
Potassium sorbate	0.5	4.0	16.0	16.0	0.5	8.0	16.0	16.0
Sodium benzoate	0.5	2.0	8.0	8.0	0.5	4.0	8.0	16.0
Sodium sulfite	0.5	0.5	1.0	2.0	0.5	0.5	2.0	2.0

MBC, minimum bactericidal concentration; MIC, minimum inhibitory concentration.

Strong antimicrobial activity (MIC = 0.5 g/L) against *Asaia lannensis* was observed at pH 4.0 in the presence of three preservatives (Table 2). Alternatively, when the pH was changed to 5, the MIC of PS, SB, and SS increased to 4 g/L, 2 g /L, and 0.5 g/L, respectively, which were much higher than the upper limits of allowable concentration (1.340, 1.180, and 0.077 g/L, respectively). These results were calculated according to the Chinese Food Safety Standards (GB2760-2014). Additionally, when the pH was 6.0, the MIC of PS, SB, and SS reached 16, 8, and 1 g/L, respectively. An increase was also observed in the MBC values. The MIC of PS and SB did not increase, while an increase was detected in the presence of SS from 1 to 2 g/L at pH 7.0 compared with pH 6.0. The results indicated that when the pH increased, the MIC and MBC values gradually increased, and the antiseptic effect of preservatives gradually decreased. Moreover, SS exhibited a higher antiseptic effect followed by SB and PS. The growth curve showed the effects of three preservatives on the growth of WLS1-1 at different pH values, and the results were consistent with those observed using the MIC (Supplementary Figure S3). Therefore, it is possible to deduce that the environmental pH could have a crucial impact on the ability of preservatives to inhibit and kill *Asaia lannensis*. In our recovered flavored syrups, the inability of PS to inhibit the growth of AS may be caused by the high pH of the environment. VBNC in WLS1-1 was induced by low temperature and preservatives.

**Figure 3:**
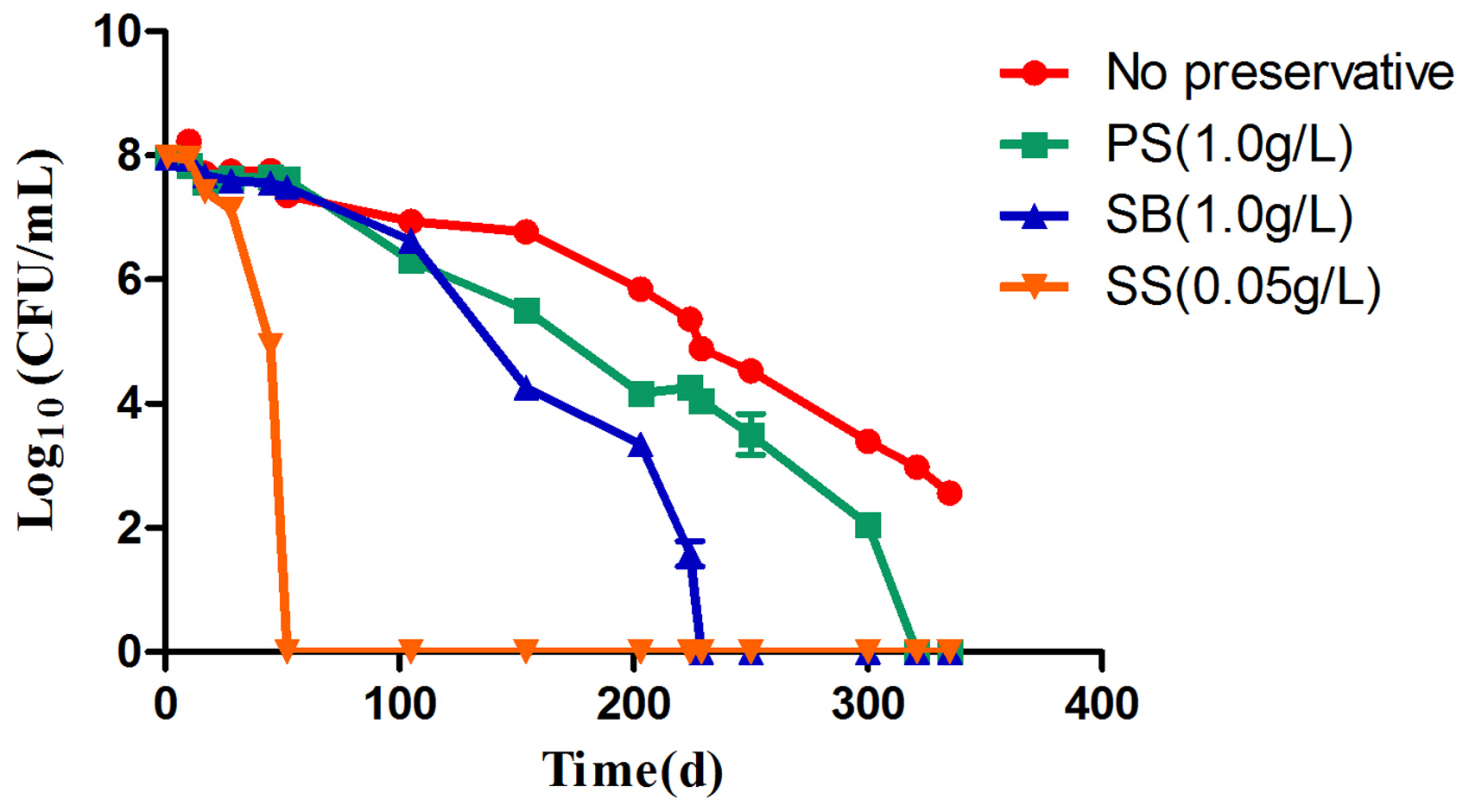
Progress curve of the culturable cell counts until the culturability was totally lost. PS: induced by potassium sorbate and 4°C; SB: induced by sodium benzoate and 4°C; SS: induced by sodium sulfite and 4°C.

**Figure 4:**
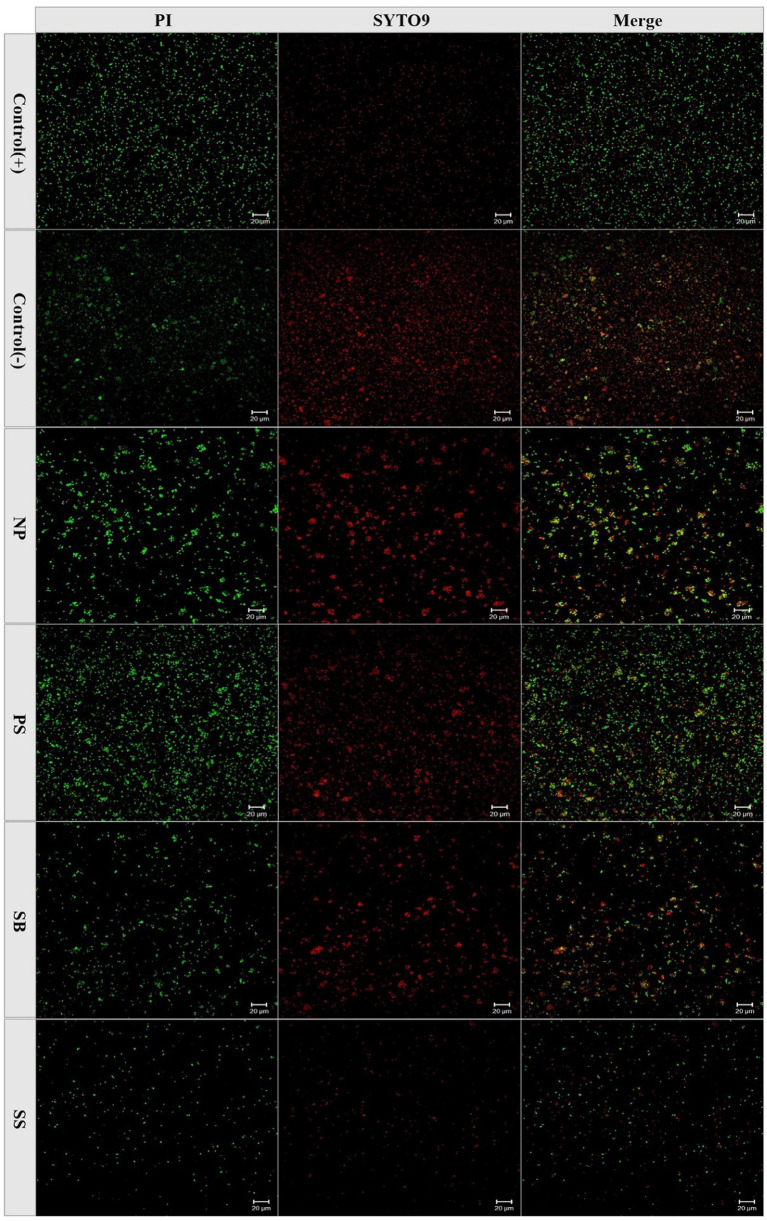
The state of WLS1-1 survival stained with PI and SYTO9. Positive control (+): non-induction; Negative control (−): 100°C for 5 min; NP: induced by 4°C; PS: induced by potassium sorbate and 4°C; SB: induced by sodium benzoate and 4°C; SS: induced by sodium sulfite and 4°C.

In the food industry, most processed food is stored at low temperatures, which range between 2 and 8°C, to extend their shelf life. Therefore, in this study, the impact of preservatives on acetic acid bacteria at a low temperature of 4°C was studied, and the results are shown in Figure 3. By increasing the induction time, the colony number gradually decreased. At 4°C, no WLS1-1 colonies were observed after 52 days of incubation with 0.05 g/L of SS and after 321 and 229 days with 1.0 g/L PS and SB, respectively. However, after 335 days, the number of colonies in WLS1-1 without preservatives decreased by more than 5 log-fold. The results showed that the WLS1-1 induced by preservatives could lose its entire culturability at 4°C, and most WLS1-1 strains only lose their culturability at 4°C. In this study, confocal laser analysis was used to observe the survival state of WLS1-1 after it had lost the ability to grow on plates (Figure 4). For this experiment, uninduced WLS1-1 was used as the positive control, and bacteria pretreated at 100°C for 5 min were used as the negative control. All the samples in the presence or absence of preservatives exhibited a large number of green-stained cells, indicating that the WLS1-1 still sustained an intact cellular structure after the loss of culturability.

### 3.3 Comparison between qPCR and PMA-qPCR to determine the VBNC WLS1-1

It has been described that standard curves can be utilized to analyze the absolute content of a target and to estimate the efficiency of qPCR. Therefore, amplification efficiency (E) close to 100% corresponds to good experimental reproducibility, while those between 90 and 110% are considered reasonable and reliable (Svec et al., 2015; Shi et al., 2022). The results demonstrated that the correlation coefficient of WLS1-1 > 0.999, and the amplification efficiency was 98.57% (Supplementary Figure S4).

**Figure 5:**
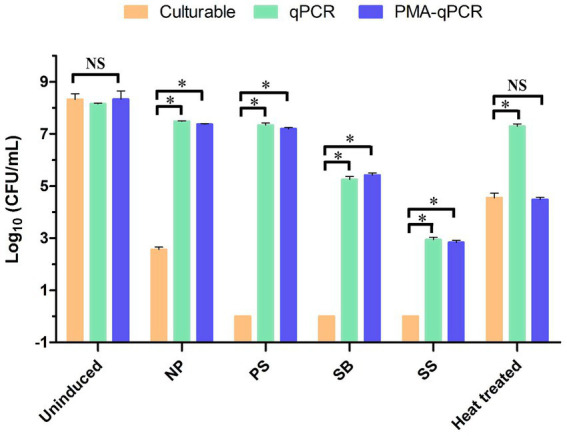
Detection of the logarithmic values of the WLS1-1 cells following different induction methods were determined by plate culture, qPCR and PMA-qPCR. *p < 0.05, NS: not significant. NP: induced by 4°C; PS: induced by potassium sorbate and 4°C; SB: inducted by sodium benzoate and 4°C; SS: induced by sodium sulfite and 4°C. PMA-qPCR, propidium monoazide-quantitative PCR; qPCR, quantitative PCR.

To determine the PMA efficiency, uninduced WLS1-1 and WLS1-1 that had been treated with 70°C heat for 5 min (to quickly produce samples that contained both live and dead bacteria) served as the control groups (Figure 5). The bacterial number of uninduced WLS1-1 was 8.321 ± 0.374, 8.153 ± 0.043 and 8.334 ± 0.556 log_10_ (CFU/mL) by the plate method, qPCR and PMA-qPCR, respectively. The number of bacteria in WLS1-1 after heat treatment was 4.550 ± 0.305, 7.287 ± 0.157 and 4.487 ± 0.139 log_10_ (CFU/mL) in the plate method, qPCR, and PMA-qPCR, respectively. The results suggest that PMA could effectively bind extracellular nucleic acids (Table 3). Additionally, for NP (no preservatives), after culture at 4°C, the number of WLS1-1 bacteria was 2.562 ± 0.168 log_10_ (CFU/mL). Furthermore, the quantitative results from qPCR and PMA-qPCR were 7.483 ± 0.035 and 7.368 ± 0.048 log_10_ (CFU/mL), respectively. Alternatively, the plate and qPCR detection methods were significantly different, which indicated that some bacteria entered into the VBNC state only after induction at 4°C. In addition, no colonies were detected in the samples treated with three preservatives combined with 4°C, and the numbers of viable bacteria detected by PMA-qPCR were 7.192 ± 0.081 (PS), 5.416 ± 0.149 (SB) and 2.837 ± 0.134 (SS) log_10_ (CFU/mL), respectively. The number of viable bacteria induced by PS was the highest, followed by SB and SS.

**Table 3:** Comparison of the number of cells by plate culturing, qPCR assays and PMA-qPCR assays on samples with different treatments.

Sample	Plate culturing log_10_ (CFU/mL)	qPCR assays log_10_ (CFU/mL)	PMA-qPCR assays log_10_ (CFU/mL)
Uninduced	8.321 ± 0.374	8.153 ± 0.043	8.334 ± 0.556
NP	2.562 ± 0.168	7.483 ± 0.035	7.368 ± 0.048
PS	0	7.334 ± 0.135	7.192 ± 0.081
SB	0	5.254 ± 0.194	5.416 ± 0.149
SS	0	2.946 ± 0.154	2.837 ± 0.134
Heat-treated	4.550 ± 0.305	7.287 ± 0.157	4.487 ± 0.139

NP: no preservatives, induced only by 4°C; PS: induced by potassium sorbate at 4°C; SB: induced by sodium benzoate at 4°C; SS: induced by sodium sulfite at 4°C; heat-treated: 70°C, 5 min. CFU, colony-forming units; PMA-qPCR, propidium monoazide-quantitative PCR; qPCR, quantitative PCR.

### 3.4 Transcriptome analysis

**Figure 6:**
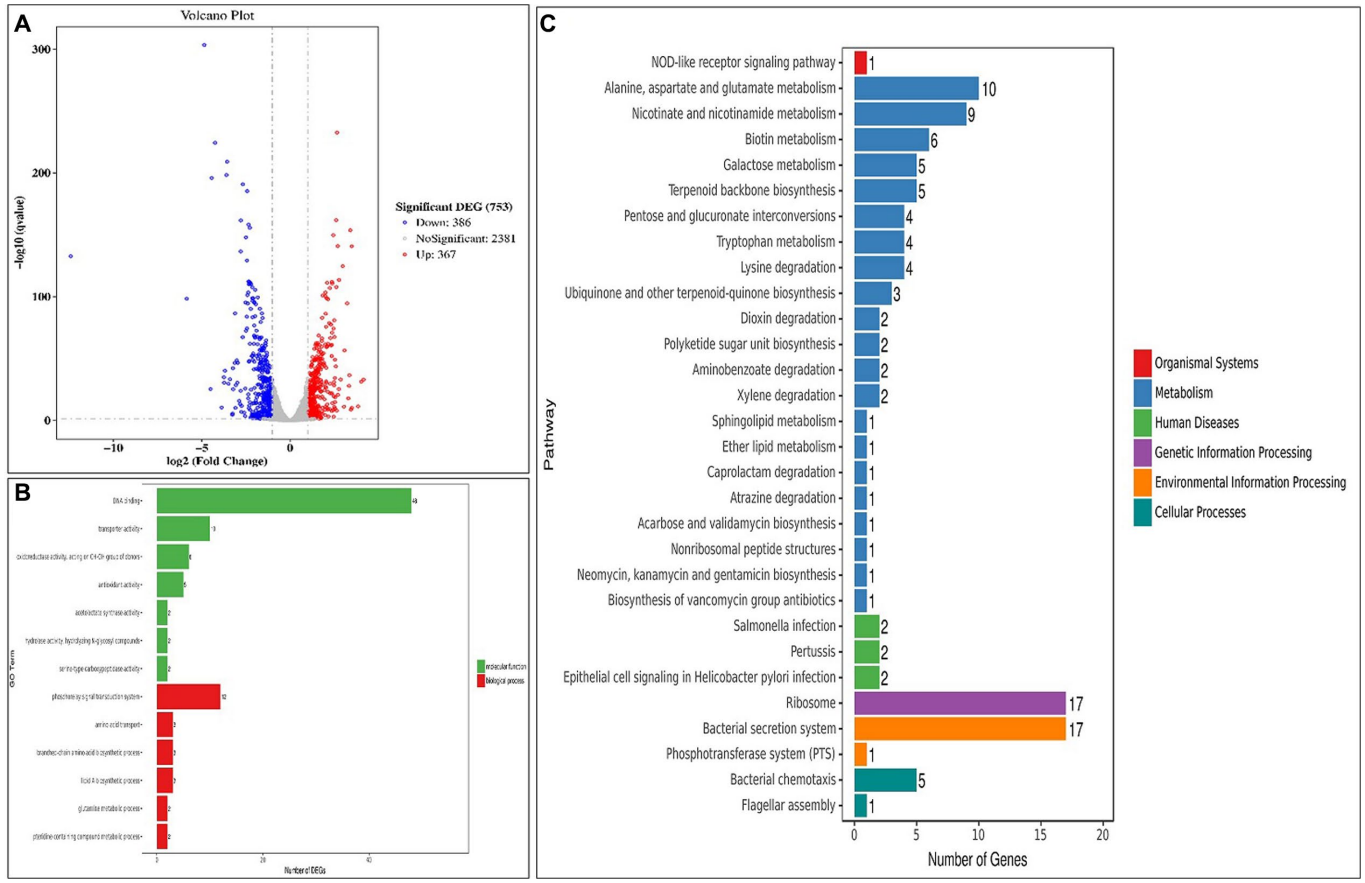
Transcriptome analysis between the normal and mixed cells (AL vs. CK). **(A)** Volcano plots of the DEGs; **(B)** Significantly enriched GO terms; **(C)** KEGG pathway analysis (top 30 DEGs). DEGs, differentially expressed genes; GO, Gene Ontology; KEGG, Kyoto Encyclopedia of Genes and Genomes.

To provide additional clarity on the mechanisms that underlie the phenotypes observed, a transcriptome analysis was conducted on the four samples. The results revealed that in the group that consisted of normal and mixed cells (AL vs. CK), 598 genes were up-regulated, and 612 were down-regulated among the DEGs (Figure 6A). Further analysis using GO enrichment demonstrated that the significantly enriched molecular function (MF) was associated with DNA binding, while the biological process (BP) was linked to the phosphorelay signal transduction system (Figure 6B). Notably, *Hup*B and *Omp*R were identified as the most significant DEGs among the down-regulated and up-regulated genes, respectively, in the GO term regulation (Supplementary Table S1).

**Figure 7:**
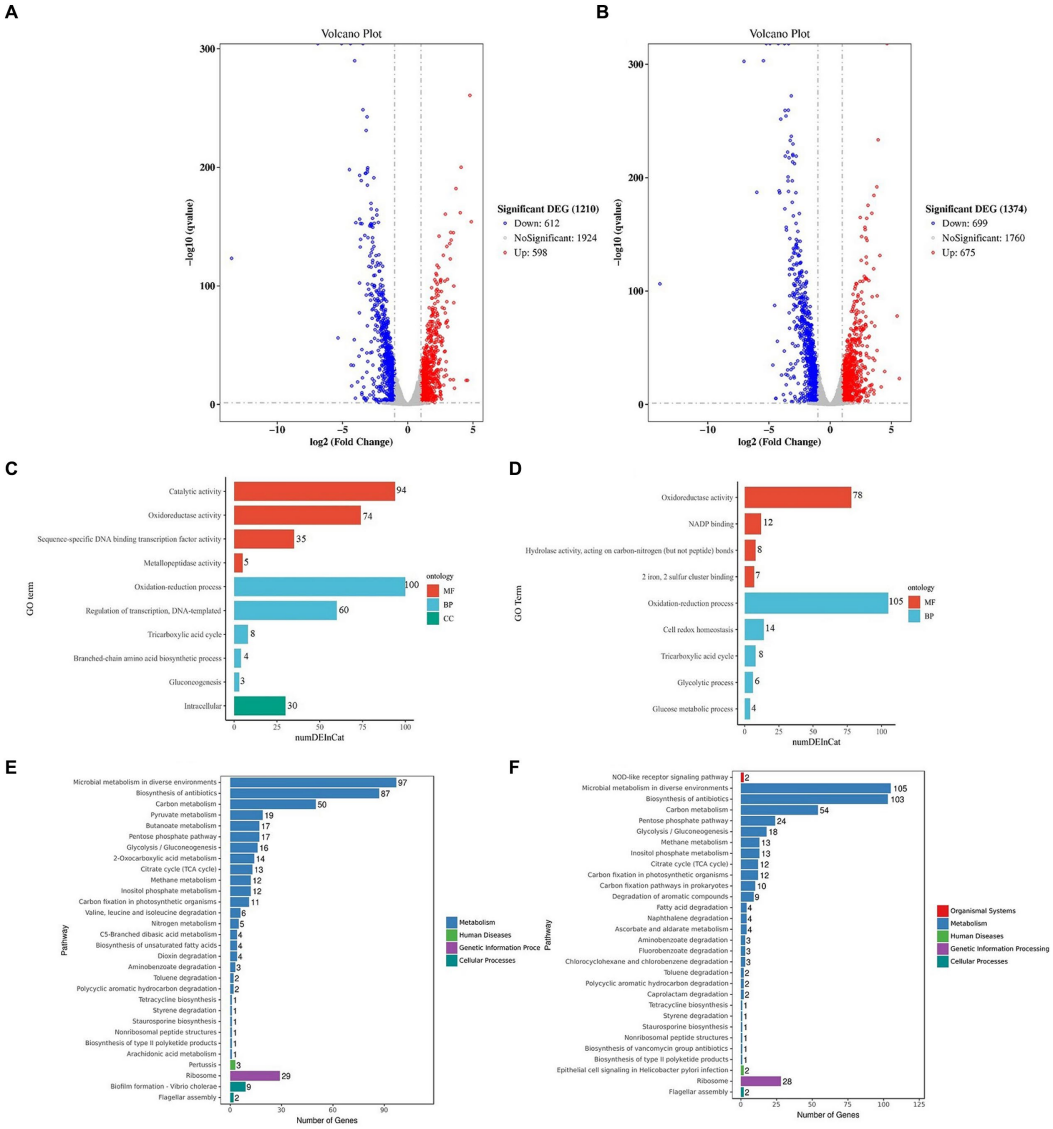
Volcano plots of the DEGs between AL and BN **(A)** and between AL and SL **(B)**. Significantly enriched GO terms of DEGs between AL and BN **(C)** and between AL and SL **(D)**. KEGG pathway enrichment of the top 30 DEGs between AL and BN **(E)** and between AL and SL **(F)**. DEGs, differentially expressed genes; GO, Gene Ontology; KEGG, Kyoto Encyclopedia of Genes and Genomes.

The KEGG analysis revealed that the ribosome and bacterial secretion system pathways exhibited the highest degree of gene enrichment, and their level 1 pathways were environmental information processing and genetic information processing, respectively (Figure 6C). The down-regulation of the ribosomal proteins is indicative of a reduction in protein synthesis by the cells, which could potentially drive them to the VBNC state (Supplementary Figure S5). Our findings also demonstrate that 14 genes were differentially expressed in the bacterial secretion system (Supplementary Figure S6), which suggested that the membrane proteins and secretory systems play an important role in the formation of the VBNC state in *Asaia lannensis*. Figures 7A,B illustrate the identification of 1,210 DEGs following induction of the VBNC state through exposure to 4°C and sodium benzoate (AL vs. BN), with 598 up-regulated and 612 down-regulated genes. In addition, 1,374 DEGs were identified through a comparison of the levels of gene expression between AL and SL, with 675 up-regulated and 699 down-regulated. To annotate the functions of the DEGs in each pairwise comparison, a GO-term function enrichment analysis was conducted independently. In the AL vs. BN group, the significantly enriched MF was involved in catalytic activity and oxidoreductase activity and BP in the oxidation–reduction process. Similar results were obtained in the AL vs. SL group (Figures 7C,D). Among the catalytic activity, the oxidoreductase activity and oxidation–reduction process, *Cys*H and *Hmp*, were the most significantly up-regulated genes, while *Gln*A, *fab*G, and *fpr* were the most significantly down-regulated genes in the three GO terms (Supplementary Tables S2, S3). The *Cys*H gene encodes 3′-phosphoadenosine 5′-phosphosulfate sulfotransferase (PAPS reductase), FAD synthetase and related enzymes, while the *Hmp* gene encodes flavodoxin reductases (ferredoxin-NADPH reductases) family 1. The protein products of *gln*A, *fab*G, and *fpr* were glutamine synthetase, SDR family oxidoreductase, and ferredoxin-NADPH reductase, respectively.

The findings indicate that *Asaia lannensis* utilizes an enzymatic antioxidant system to sustain the intracellular reducing environment during the formation of the VBNC state through a combination of preservatives and low temperature. Additionally, the KEGG analysis identified the top 30 significant pathways, with three main metabolic pathways that were linked to the bacterial survival state, namely microbial metabolism in diverse environments, carbon metabolism, and ribosome in AL vs. BN and AL vs. SL (Figures 7E,F). In the metabolism pathway, the genes were significantly enriched in the pentose phosphate pathway and citrate cycle (TCA cycle) pathway (Supplementary Figure S7). Within the genetic information processing pathway, there was a significant enrichment of the genes associated with the ribosome (Supplementary Figures S8, S9), as supported by the results of the enrichment analysis in AL vs. CK. However, a greater number of genes were implicated in the regulation of the ribosome metabolic pathway, which indicated a further reduction in protein synthesis under the influence of preservatives. The observed downregulation of the pentose phosphate pathway, a process of glucose oxidative decomposition, suggests that the bacteria are unable to metabolize glucose in their environment. Additionally, the reduced expression of the TCA pathway provides further evidence of the inactivity of VBNC bacteria.

## 4 Discussion

### 4.1 Detection and analysis of the source of *Asaia lannensis* in food

In the food industry, the detection of microorganisms is usually performed by traditional plate culture methods. However, some microorganisms that can tolerate exposure to salt, sugar, heat, and cold and environmental stress, such as starvation, high pressure, extreme temperature, hypoxia, and fungicides, could not be isolated using this traditional method (Dong et al., 2020; De los Ángeles Rey et al., 2022). Some foods tested for microorganisms still spoiled after shelf storage with thermostable *Lactobacillus* (Liu et al., 2018) and *Campylobacter* (Wulsten et al., 2022). Therefore, it is difficult to prevent and control these types of bacteria that can spoil food.

*Asaia lannensis*, isolated from contaminated flavored syrups, can be found in plants and flowers and is an uncommon contaminant in perishable soft drink organic matter, such as fruit-flavored bottled water, fruit drinks and iced tea, and strawberry-flavored bottled mineral water. In this study, acetic acid bacteria isolated from high-sugar flavored syrups could not grow in conventional media. However, when the sugar content and the temperature were changed from 36 to 28°C, there was significant growth in the acetic acid bacteria. Moreover, the presence of few colonies indicates that *Asaia lannensis* is a glycophilic species that can adapt to different environments (Fernandez et al., 2011).

Since flavorful syrups are composed of a large quantity of honey, this microorganism could be transported into the honey by bees after picking nectar. Therefore, incomplete disinfection during the honey processing could induce contamination with *Asaia lannensis*. Furthermore, another possible source of contamination is the presence of insects in the raw material or during the production process since *Asaia* sp. is an internal parasite present in *Drosophila* and *Plasmodium* (Chouaia et al., 2010; Crotti et al., 2010; Deutscher et al., 2018).

### 4.2 Increased resistance of *Asaia lannensis* to food preservatives

Over the years, preservatives have been added to food to control the growth of microorganisms and extend the shelf life. It has been described that microorganisms can become resistant to preservatives when exposed for a long time. Therefore, exposure to antimicrobials, such as SB and PS, in sublethal concentrations can lead to adaptation by the microorganisms. In fact, *Sporolactobacillus* has been shown to grow at levels of potassium sorbate below 7,000 mg/L and sodium nitrite below 2,000 mg/L (Botha and Holzapfel, 1987). The strains of *Lactobacillus* that have been studied were virtually uninhibited by sorbate levels up to 1,000 ppm (Edinger and Splittstoesser, 1986). Previous studies showed that *Asaia* sp. was isolated from the reclaimed fruit beverages, and a reasonable degree of inhibition was identified when the concentration of sorbate and benzoic acid was as high as 7 mmol/L (near 1,000 mg/L) (Horsáková et al., 2009). In this study, there were inhibitory effects on the growth of *Asaia lannensis* at levels of potassium sorbate, sodium benzoate, and sodium sulfite up to 16 g/L, 16 g/L, and 2.0 g/L, respectively, which showed that *Asaia lannensis* was strongly resistant to the three preservatives. Additionally, osmotolerant microorganisms are crucial for developing contamination in low pH foods and beverages with high contents of sugar (Thomas and Davenport, 1985). These microorganisms can adapt their growth in the presence of high concentrations of preservatives (higher than those allowed legally in foodstuffs) (Cole and Keenan, 1986; Warth, 1988). This study demonstrated that the effect of preservatives on *Asaia lannensis* was highly dependent on pH (Figure 5) since the inhibition of cells was only detected in the presence of a lower pH value (pH < 5.0). Alternatively, the MIC values of SB, PS, and SS were higher than the recommended effective concentrations (GB2760-2014). This indicates that allowable concentrations of food preservatives have no antibacterial effects on *Asaia lannensis*, which increases the possibility of food spoiling.

The pH of a food can alter the effectiveness of an antimicrobial compound. Organic acids are most effective in their undissociated form. The concentration of the undissociated acid is dictated by the food pH and pKa of the acid (Taylor et al., 2020). The pKa for benzoic acid is 4.19, and that of sorbic acid is 4.76. A food pH that is below the pKa of the particular acid shifts the equilibrium toward the undissociated form. An example is benzoic acid versus benzoate. The undissociated form has a higher efficacy toward microorganisms. Therefore, weak acid preservatives are more effective in the low pH range. Our research has also confirmed this conclusion. However, some compounds based on organic acids can be active at low concentrations in food products with a pH ≥ 5.5. In this study, 1.0 g/L of PS could not inhibit the growth of *Asaia lannensis* at pH values > 5.0. This might be one of the main reasons for the spoilage of flavored syrup.

### 4.3 *Asaia lannensis* could be induced to enter the VBNC state by low temperatures and preservatives

Many industrial manufacturers, including those of food, drugs, and cosmetics, often need to explain why there are no microorganisms in products that contain preservatives during their routine monitoring, but outbreaks and growth still occur after a period of storage. This phenomenon could be explained because microorganisms can enter the VBNC state and become indetectable by traditional methods. During this process, some microorganisms are destroyed, but others will survive and enter the VBNC state, which subsequently causes food spoilage or poisoning. Therefore, low-temperature refrigeration is the most common strategy to preserve and control food quality. However, some studies have demonstrated that low contents of nutrients and low temperature are the main causes of VBNC induction in pathogenic bacteria (Mizunoe et al., 2000; Besnard et al., 2002; Su et al., 2013).

To our knowledge, this study is the first to observe that acetic acid bacteria can enter the VBNC state under conditions of a combination of preservatives and low temperatures. Previous studies demonstrated that PS could promote the VBNC state in some microorganisms. One study showed that *Escherichia coli* could enter the VBNC state after 15 days in the presence of sorbate (10 g/L) (Ogane et al., 2019). Zhong et al. (2018) revealed that *Vibrio parahaemolyticus* ATCC 17802 could enter the VBNC state after 40 days at 4°C in seawater that contained 10 mmol/L (1.5 g/L) of PS. Furthermore, when *L. monocytogenes* were treated with 50 mM PS, the cells could enter the VBNC state for several hours at 37°C and pH 4.0 (Cunningham et al., 2009). However, the mechanism of the VBNC state induced by PS, SB, and SS remains unclear. Some research has demonstrated that these acidic preservatives could increase the ability of microorganisms to adapt to a stressful environment (He et al., 2022). Overall, the results of this study suggest that bacteria in the presence of preservatives have a higher probability of entering the VBNC state than those without preservatives, which indicates that preservatives could enhance the response of bacteria to stress.

The transcriptomic data explained the differences in the level of expression of the genes involved in the formation of VBNC state under different induction conditions. Compared with the normal group, the level of expression of the gene that regulates the response to oxidative stress was significantly up-regulated in low temperature induction (*Hup*B and *Omp*R), and low temperatures combined with preservatives induced the VBNC cells (*Cys*H, *Hmp*, *gln*A, *fab*G, and *fpr*). The product of the *Hup*B gene is a DNA-binding protein that mediates stress responses (Hudson and Ortlund, 2014; Singh et al., 2022). Additionally, the *Omp*R family is a component of the regulatory network that controls the oxidative stress response (Zhao et al., 2019). These findings suggest that low temperatures can enhance the responses of bacteria to stress, which can potentially result in the formation of the VBNC state. Indeed, previous research has shown that entry into the VBNC state is a response to oxidative stress (Liu et al., 2016; Liao et al., 2021). Our results led us to hypothesize that cells in the VBNC state activate defense mechanisms against oxidative stress. This includes an increase in the biosynthesis of the intracellular gene *Cys*H, which encodes a catalytic enzyme that can directly repair the primary structure of certain covalently modified proteins. One of the most prevalent modifications is the reductive oxidation of disulfide bonds. Notably, the transfer of electrons from NADPH to thioredoxin via the flavin carrier, as exemplified by the significant changes in *Hmp* and *fpr* genes that encode the flavodoxin reductases observed in this study, plays a crucial role in this process. The accumulation of genes that are implicated in oxidative stress response mechanisms appears to be a fundamental survival strategy employed by the *Asaia lannensis* community in response to exposure to low temperatures and preservatives. Furthermore, the KEGG metabolic pathway analysis revealed that induction of the VBNC bacteria by three preservatives (CK, BN and SL) was notably enriched in the ribosome pathway, which governs the processing of genetic information. In particular, the EF-Ts protein facilitates the regeneration of the EF-Tu-GDP complex into its active form, EF-Tu-GTP, while EF-Tu regulates translation by interacting with the tRNA and ribosomes, thereby inhibiting the translation of superfluous proteins and triggering the biosynthesis of stress-induced proteins (Young and Bernlohr, 1991).

This study confirms previous research by Heim et al. (2002) and Jia et al. (2013) of the downregulation of EF-Tu and EF-Ts in response to the VBNC state in *Enterococcus faecalis* and *Vibrio harveyi*, respectively. These findings suggest that EF-Tu and EF-Ts may be involved in the regulation of cell growth and stress responses. The results of a transcriptional analysis provide insight into why the bacteria were more prone to enter the VBNC state when exposed to low temperatures and preservative for induction as opposed to low temperature alone. In the VBNC cells induced at 4°C, the differential gene functions were primarily associated with DNA binding and the phosphorelay signal transduction system, and the metabolic pathway was focused on the ribosome and bacterial secretion system. This regulatory mechanism was also observed in the VBNC state of *E. faecalis* induced at 4°C (Heim et al., 2002). However, the combination of low temperatures and treatment with preservatives resulted in differences in gene expression that were concentrated in the catalytic activity, oxidoreductase activity and oxidation–reduction process. The metabolic pathway was centered on the regulation of cellular metabolic activity, which suggested that the addition of preservatives enhanced the regulation of cell resistance to oxidation–reduction reactions, and the metabolic activity decreased significantly. In addition, the cells induced by SB (229 d) entered the VBNC state earlier than those induced by PS (321 d). A GO enrichment analysis revealed that 94 genes in the AL vs. BN group were enriched in catalytic activity in MF, while 60 genes were enriched in the regulation of transcription and DNA templated; 30 genes were enriched in intracellular in the CC with no enrichment observed in the AL vs. SL group (Figure 7). These findings suggest that SB may promote the expression of a greater number of differential genes involved in the regulation of transcription, which led to an earlier induction of the VBNC state in cells.

## 5 Conclusion

In this study, *Asaia lannensis* was isolated and identified in flavored syrup that had spoiled. These microorganisms can thrive in media with a high content of sugar. Additionally, the bacteria were resistant to commonly utilized food preservatives, such as PS, SB, and SS. The findings of this study also revealed that there was a significant reduction in antibacterial activity of the preservatives at pH ≥ 5. *Asaia lannensis* can enter into the VBNC state at 4°C and in the presence of sublethal concentrations of these preservatives at 4°C. The transcriptome analysis indicated that the formation of VBNC cells was primarily attributed to the oxidative stress induced by low temperature and preservatives. In parallel, the cells exhibited a strategy for survival by downregulating non-essential protein synthesis and metabolic activity. Thus, it is crucial for food manufacturers and quality supervision departments to exercise the utmost caution owing to the resistance of *Asaia lannensis* toward preservatives and its ability to enter the VBNC state. This study aids in understanding the potential origins of food contamination caused by *Asaia lannensis*. Furthermore, it can provide a theoretical basis for future research efforts, including the examination of factors that induce *Asaia lannensis* to enter the VBNC state through proteomics and metabolomics, as well as the development of rapid resuscitation techniques to accurately detect the bacteria and ultimately efficaciously manage them.

## Data availability statement

The datasets presented in this study can be found in online repositories. The names of the repository/repositories and accession number(s) can be found in the article/Supplementary material.

## Author contributions

XW: Data curation, Formal analysis, Investigation, Methodology, Software, Visualization, Writing – original draft. YC: Writing – original draft, Methodology, Visualization. SZ: Methodology, Data curation, Writing – original draft. A-tS: Data curation, Methodology, Writing – original draft. DH: Visualization, Writing – original draft. GZ: Visualization, Writing – original draft. XX: Project administration, Supervision, Writing – review & editing. JW: Conceptualization, Funding acquisition, Project administration, Supervision, Writing – review & editing, Validation.
