# Supplementary material for: Resistance to preservatives and the viable but non-culturable state formation of Asaia lannensis in flavored syrups
Source: Front Microbiol. 2024 Feb 16;15:1345800. doi: 10.3389/fmicb.2024.1345800 (PMC10904602; doi:10.3389/fmicb.2024.1345800)
Supplement: Supplementary file 1 [file Data_Sheet_1.ZIP › supplementary material/Supplementary material_Resistance to preservatives and the Viable but Non-culturable (VBNC) State Formation of spoilage bacteria in flavored syrups.docx]

Resistance to preservatives and the Viable but Non-culturable (VBNC) State Formation of spoilage bacteria in flavored syrups

Xia Wen^1,2,3^, Yiwen Chen^3^, Shuyao Zhang^3^, Aiting Su^3^, Di Huang^3^, Gang Zhou^3^, Xiaobao Xie^3*^, Jufang Wang^1,2*^

^1^School of Biology and Biological Engineering, South China University of Technology, Guangdong, 510006, China

^2^Guangdong Provincial Key Laboratory of Fermentation and Enzyme Engineering, South China University of Technology, Guangzhou 510006, China

^3^Key Laboratory of Agricultural Microbiomics and Precision Application (Ministry of Agriculture and Rural Affairs), Guangdong Provincial Key Laboratory of Microbial Culture Collection and Application, Key Laboratory of Agricultural Microbiome (Ministry of Agriculture and Rural Affairs), State Key Laboratory of Applied Microbiology Southern China, Guangdong Detection Center of Microbiology, Institute of Microbiology, Guangdong Academy of Sciences, Guangzhou ,510070, Guangdong, China

***Correspondence:**Corresponding Author

[xiaobaoxie@126.com](mailto:xiaobaoxie@126.com)

[jufwang@scut.edu.cn](mailto:jufwang@scut.edu.cn)

Supplementary Material

# Supplementary Figure and Tables

**Figure S1:** The samples of RNA-seq transcriptomics: AL: uninduced *Asaia lannaensis*; ①CK: sample subjected to procedure (4℃ induction); ②BN: sample after procedure (4℃+1.0g/L sodium benzoate induction); ③ SL: Sample subjected to procedure (4℃+1.0g/L potassium sorbate induction)

**Figure S2:** Phylogenetic tree of *Asaia* and related strains constructed based on 16S rRNA gene sequences. Phylogenetic tree based on 16S rRNA gene sequences of strains WLS1-1(mark in black triangle symbol) and representatives of all currently validly described species of the genus *Asaia* inferred using the NJ method. Gaps and missing data were excluded. The optimal tree with the sum of branch length of 0.31395809 is shown. Accession numbers are shown in brackets. Bootstra pvalues (≥50%) based on 1000 iterations are shown as percentages at the nodes. Bar, 0.01 nucleotide substitutions per site. Evolutionary analyses were conducted in mega X.

**Figure S3**: The growth curves of WLS1-1 strains in the presence of different pH and concentrations of preservatives. A: Potassium sorbate; B: Sodium benzoate; C: Sodium sulfite

**Figure S4:** Standard curve of viable WLS1-1by qPCR.

**Figure S5**: The significantly differentially expressed in ribosomal in AL vs CK. Red(up-regulated) and blue(down-regulated).

**Figure S6**: The significantly differentially expressed in the bacterial secretion system in AL vs CK. Red(up-regulated) and blue(down-regulated).

**Figure S7**: The metabolic pathways of microbial metabolism in diverse environments(**A**) and carbon metabolism(**B**) in AL vs BN and AL vs SL. Red(up-regulated) , blue(down-regulated) and green(up-regulated and down-regulated).

**Figure S8**: The significantly differentially expressed in the metabolic pathways of ribosomal in AL vs BN. Red(up-regulated) and blue(down-regulated).

**Figure S9**: The significantly differentially expressed in the metabolic pathways of ribosomal in AL vs SL. Red(up-regulated) and blue(down-regulated).

## Supplementary Figures


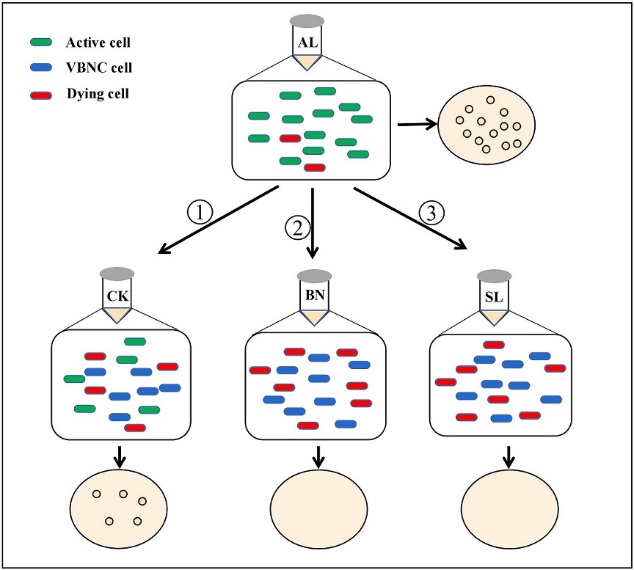


**Figure S1:** The samples of RNA-seq transcriptomics: AL: uninduced *Asaia lannaensis*; ①CK: sample subjected to 4℃ induction; ②BN: sample after procedure (4℃+1.0g/L sodium benzoate induction); ③ SL: Sample subjected to procedure (4℃+1.0g/L potassium sorbate induction)


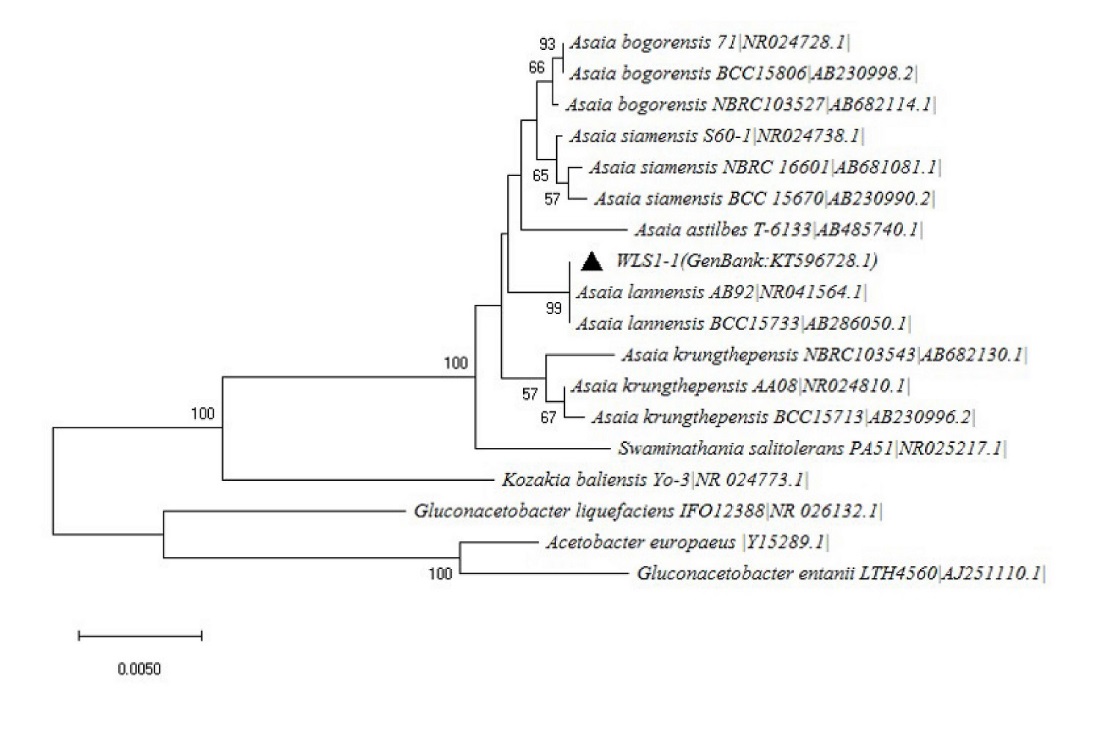


**Figure S2:** Phylogenetic tree of *Asaia* and related strains constructed based on 16S rRNA gene sequences. Phylogenetic tree based on 16S rRNA gene sequences of strains WLS1-1(mark in black triangle symbol) and representatives of all currently validly described species of the genus *Asaia* inferred using the NJ method. Gaps and missing data were excluded. The optimal tree with the sum of branch length of 0.31395809 is shown. Accession numbers are shown in brackets. Bootstra pvalues (≥50%) based on 1000 iterations are shown as percentages at the nodes. Bar, 0.01 nucleotide substitutions per site. Evolutionary analyses were conducted in mega X.


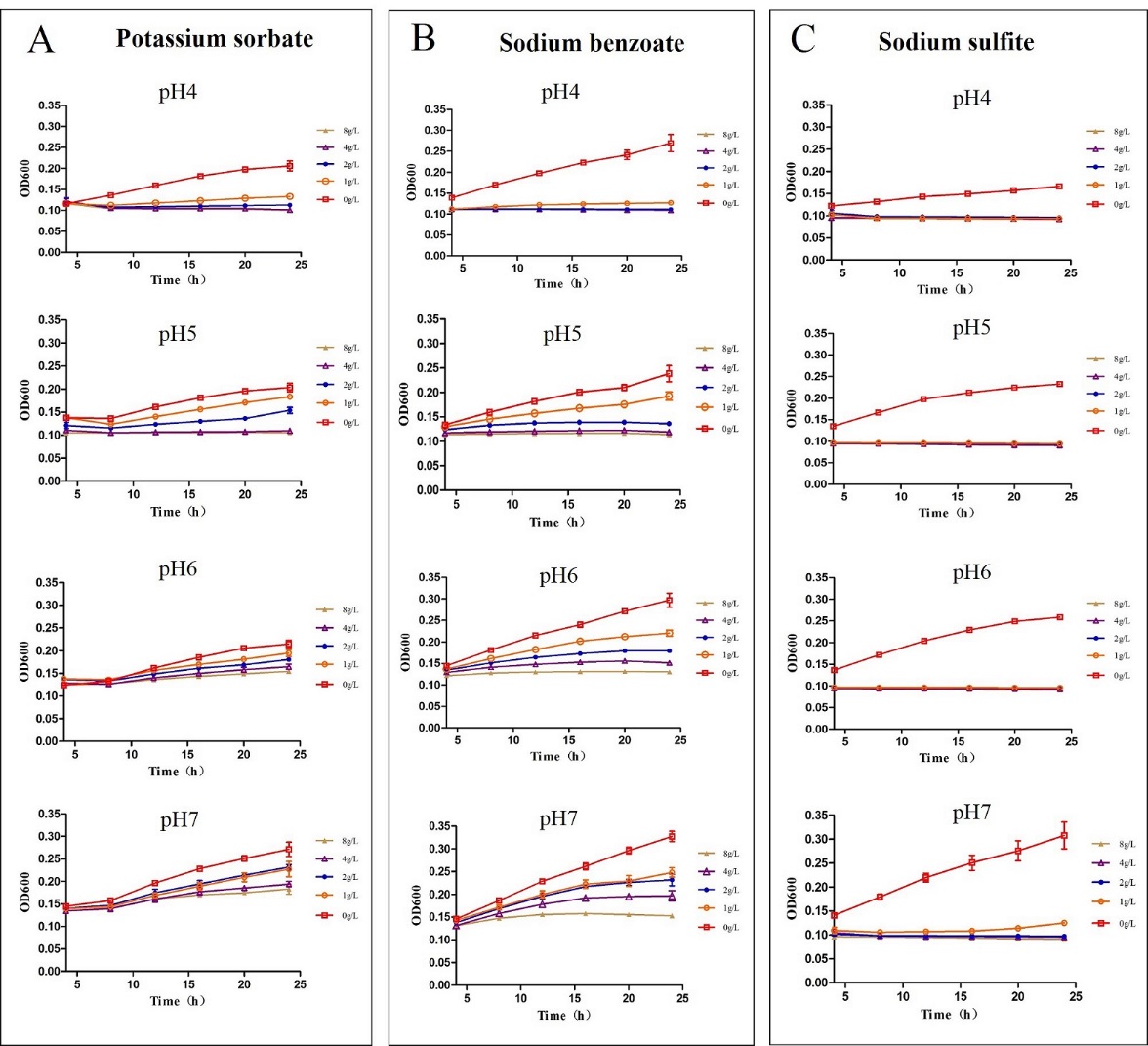


**Figure S3**: The growth curves of WLS1-1 strains in the presence of different pH and concentrations of preservatives. A: Potassium sorbate; B: Sodium benzoate; C: Sodium sulfite.


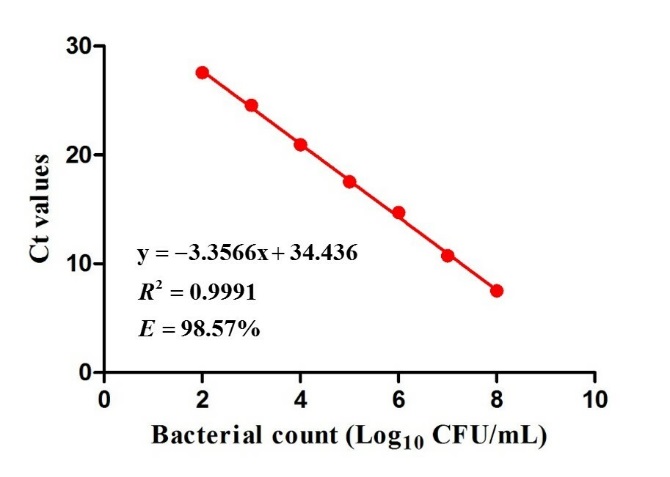


**Figure S4:** Standard curve of viable WLS1-1by qPCR.


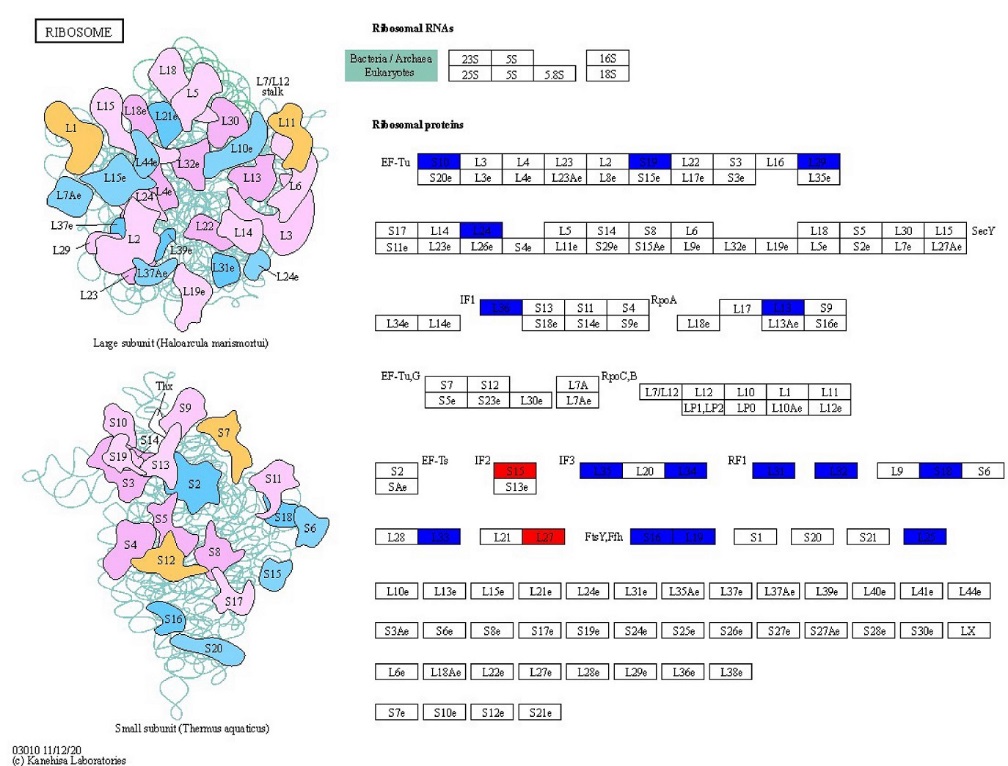


**Figure S5:** The significantly differentially expressed in ribosome in AL vs CK. Red (up-regulated) and blue(down-regulated).


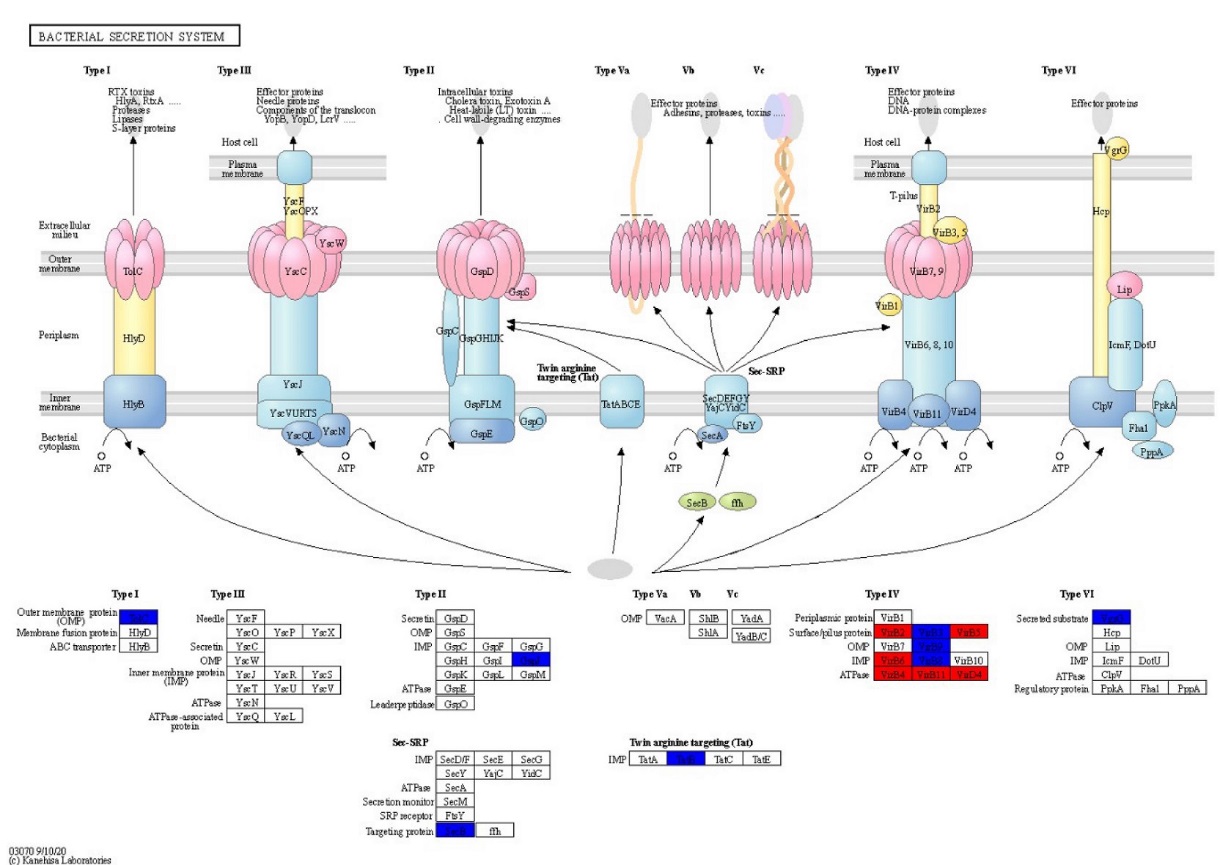


**Figure S6:** The significantly differentially expressed in the bacterial secretion system in AL vs CK. Red(up-regulated) and blue(down-regulated).


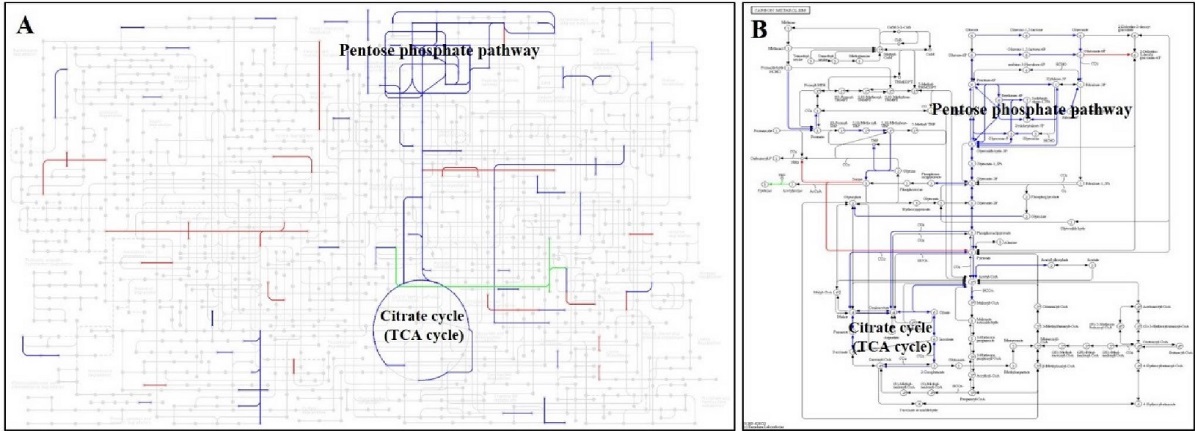


**Figure S7**: The metabolic pathways of microbial metabolism in diverse environments(**A**) and carbon metabolism(**B**) in AL vs BN and AL vs SL. Red(up-regulated) , blue(down-regulated) and green(up-regulated and down-regulated).


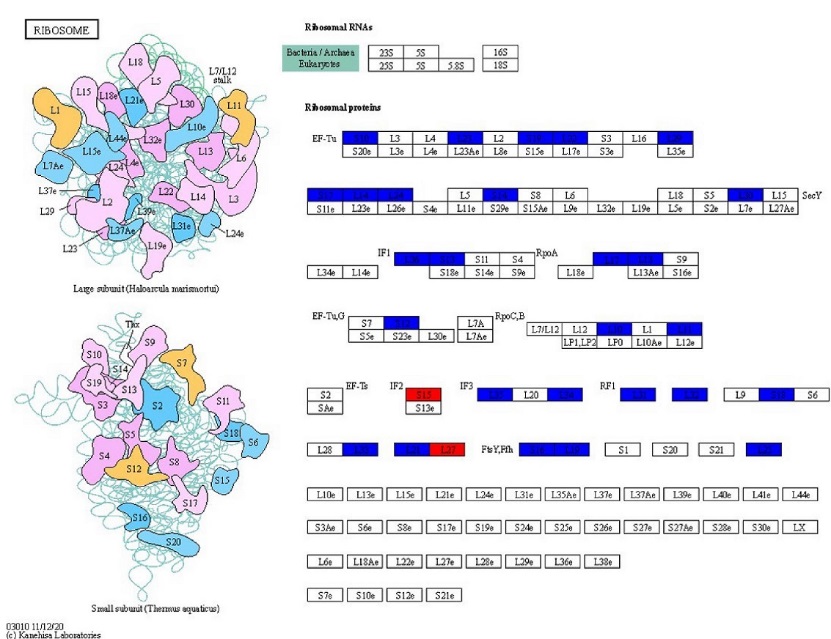


**Figure S8**: The significantly differentially expressed in the metabolic pathways of ribosome in AL vs CK. Red (up-regulated) and blue(down-regulated).


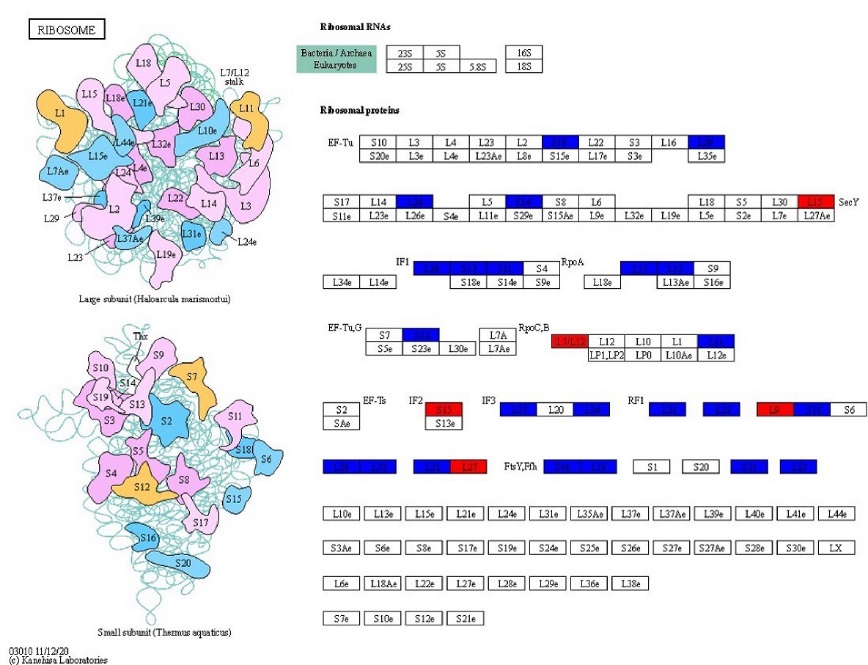


**Figure S9**: The significantly differentially expressed in the metabolic pathways of ribosome in AL vs SL. Red (up-regulated) and blue(down-regulated).
